# Traumatic Acid Reduces Oxidative Stress and Enhances Collagen Biosynthesis in Cultured Human Skin Fibroblasts

**DOI:** 10.1007/s11745-016-4174-5

**Published:** 2016-07-16

**Authors:** Agata Jabłońska-Trypuć, Walentyn Pankiewicz, Romuald Czerpak

**Affiliations:** 1 Faculty of Civil and Environmental Engineering, Division of Sanitary Biology and Biotechnology, Bialystok University of Technology, Wiejska Street 45E, 15-351 Białystok, Poland; 2 University of Medical Science in Białystok, Krakowska Street 9, 15-875 Białystok, Poland

**Keywords:** Traumatic acid, Cytokinins, Collagen, Glutathione peroxidase, Glutathione reductase, Catalase, Lipid peroxidation, Glutathione

## Abstract

Traumatic acid (TA) is a plant hormone (cytokinin) that in terms of chemical structure belongs to the group of fatty acids derivatives. It was isolated from *Phaseolus vulgaris*. TA activity and its influence on human cells and organism has not previously been the subject of research. The aim of this study was to examine the effects of TA on collagen content and basic oxidative stress parameters, such as antioxidative enzyme activity, reduced glutathione, thiol group content, and lipid peroxidation in physiological conditions. The results show a stimulatory effect of TA on tested parameters. TA caused a decrease in membrane phospholipid peroxidation and exhibited protective properties against ROS production. It also increases protein and collagen biosynthesis and its secretion into the culture medium. The present findings reveal that TA exhibits multiple and complex activity in fibroblast cells *in vitro*. TA, with its activity similar to unsaturated fatty acids, shows antioxidant and stimulatory effects on collagen biosynthesis. It is a potentially powerful agent with applications in the treatment of many skin diseases connected with oxidative stress and collagen biosynthesis disorders.

## Introduction

Traumatic acid (TA, trans-2-dodecenedioic acid), which is an oxidative derivative of unsaturated fatty acids, was isolated in 1937 by Bonnier and English from *Phaseolus vulgaris*. It was proved that TA is quite common in mesophyl and meristem tissues in many plant species. Traumatic acid and its aldehyde derivative—traumatin (2-dodeceno-1-al-10-carboxylic acid)—are called wound hormones, because they appear in relatively high amount around wounds and they stimulate cells division. Their presence is detected especially in young, intensively growing organs like: leafs, fruits and seeds. In selected plant species it was shown that traumatin’s biological activity is higher than traumatic acid’s [1, 2].

**Fig. 1:**
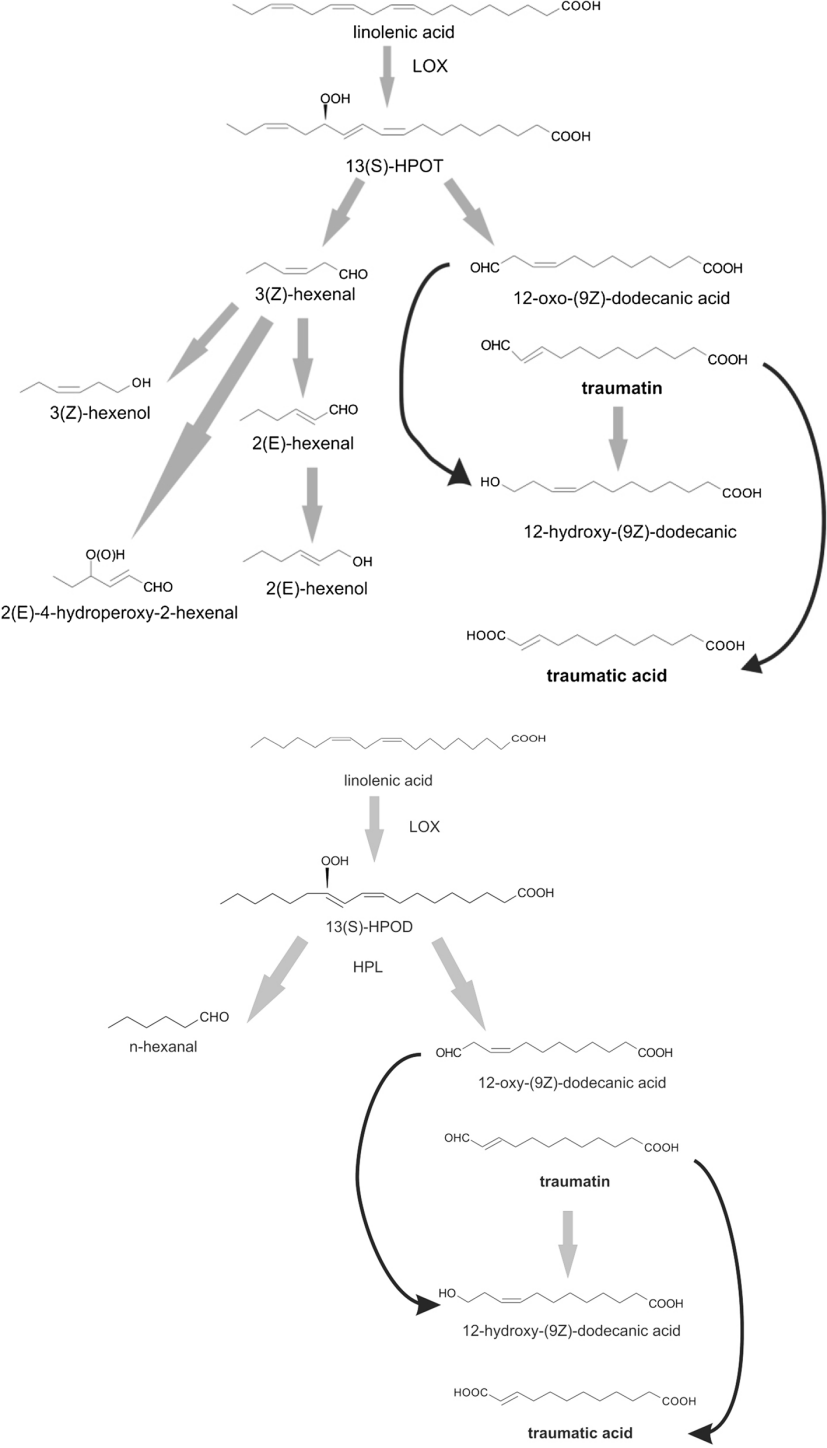
Traumatic acid biosynthesis

TA biosynthesis may start either from linoleic acid or linolenic acid. These are both 18-carbon unsaturated fatty acids precursors (Fig. 1). Due to the activity of wound-inducible phospholipases A2 and D, linoleic and linolenic acid are released from the lipids that are present in cell membranes. The next stage in this process is lipooxygenase (LOX)-catalyzed oxidation. The presence of LOX (EC 1.13.11.12) was detected in all eukaryotic cells. Its main activity is catalyzing the addition of the oxygen molecule to the structure of unsaturated fatty acids, which is the first step in traumatic acid biosynthesis in plants. This LOX-catalyzed reaction's major products are 13-hydroperoxylinolenic acid (13HPOT) and 13-hydroperoxylinoleic acid (13HPOD), which then undergo a reaction catalyzed by hydroperoxide lyase (HPL). 18-Carbon chains of 13-HPOT or 13-HPOD are cleaved into two 6-carbon chain aldehydes: (2E)-hexenal and hexanal and 12-carbon 12-oxo-(9Z)-dodecanic acid. After isomerization, 12-oxo-(9Z)-dodecanic acid is converted into 12-oxo-(10E)-dodecanic acid, or otherwise called traumatin. Subsequently, as a result of nonenzymatic autooxidation of traumatin, traumatic acid is synthetized [3–6].

In all probability, TA's main activity in plants is growth stimulation and the induction of cell proliferation and elongation, although it acts not as strongly as it has been observed in the case of traumatin [7, 8]. In *Chlorella vulgaris* culture, the addition of TA caused a twofold increase in algal cell number as compared to the control. However, the molecular mechanism of TA action at the cellular level is still not known and there is a significant lack of literature data concerning this agent's influence on basic metabolic processes. Researches conducted on unicellular green alga *Chlorella vulgaris* demonstrated TA's positive influence on: chlorophyll a and chlorophyll b content, carotenoid content, water-soluble protein content and monosaccharide content. According to Asafova *et al*. [7] TA stimulates tyrosine phosphorylation in proteins at least 1.5-fold. The increase of phosphorylated tyrosine residues content was the highest for proteins with molecular mass, e.g., 19, 20, 22, 26, 31, 42 and 74 kDa. After TA stimulation, macropeptides with molecular mass 36, 47 and 49 kDa showed the inhibition of tyrosine phosphorylation. Most likely the plant hormone may influence protein kinase and phosphatase activity by the intensification or inhibition of tyrosine phosphorylation in proteins. It is possible that after TA binding to a specific receptor on a cell membrane, appropriate kinases are activated. Sequential steps are phosphorylation and conformational changes in protein structures and activation of a signal transduction pathway leading to modifications in expression of specific genes [7].

**Fig. 2:**
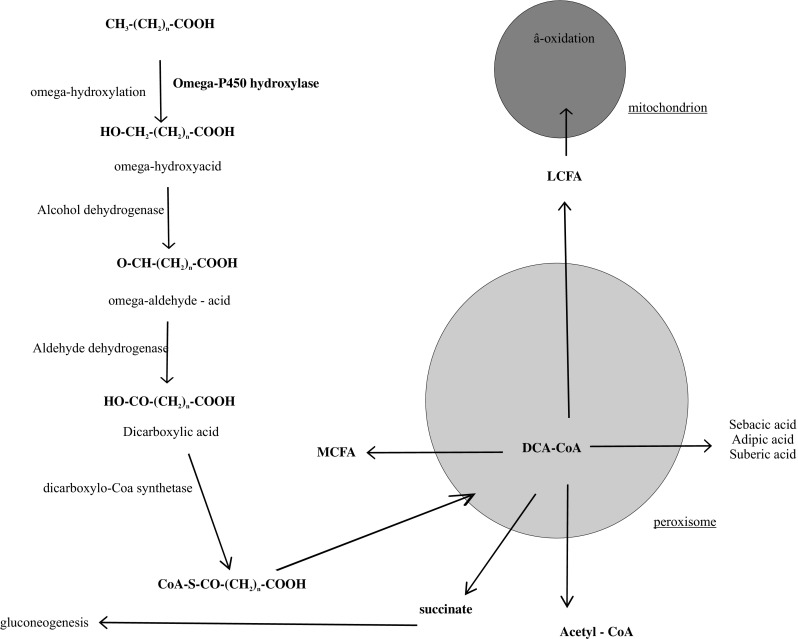
Fatty acids omega oxidation

The ω-oxidation process in animal cells occurs in an analogous manner to TA biosynthesis in plants (Fig. 2). The first step in this process is ω-hydroxylation, in which cytochrome P-450, NADPH_2_ and O_2_ participate. As a result of the conversion of a CH_3_ group to a CH_2_OH group and subsequently its oxidation through the aldehyde group (–CHO) to –COOH, dicarboxylic acid arises. The ω-hydroxylation process is mediated by cytochrome P450 and both saturated and unsaturated branched-chain fatty acids subject to this process. Many of the physiologically important compounds such as arachidonic acid, prostaglandins and leukotrienes undergo metabolic changes through the ω-hydroxylation process. Dicarboxylic acids formed by the action of two cytosolic enzymes, alcohol dehydrogenase and aldehyde dehydrogenase, are subsequently oxidized by the peroxisome β-oxidation system. Several cycles of the β-oxidation system in peroxisomes produce shorter-chain fatty acids. These shorter-chain fatty acids can undergo three processes: firstly, after metabolic conversion to sebacic, adipic and suberic acid, they can be excreted from the excretory system as a components of urine; secondly, they can undergo complete oxidation by the mitochondria β-oxidation system, and thirdly, they can serve as a source of succinate, which is a precursor in gluconeogenesis, and acetyl CoA. In the liver, acetyl CoA is metabolized to acetate which subsequently can be a lipogenic precursor for fatty acids and cholesterol synthesis. An acetate may also be a potential source of energy for peripheral tissues. In conclusion, it must be noted that the ω-hydroxylation process is of minor importance in the metabolism of fatty acids (4–15 %); however, its value significantly increases if the following happens: starvation, alcohol abuse, hydrolipidemic drugs treatment and various metabolic diseases [9–12].

In vivo studies have revealed that both mitochondria and peroxisomes are connected with the catabolism of dicarboxylic acids. The role of mitochondria appears to be tissue- and substrate-dependent and presumably is determined by carnitine dicarboxyltransferase activity. It was demonstrated that peroxisomes can only shorten dicarboxylic acids, because the peroxisomal β-oxidation system cannot maintain dicarboxylic acids shorter than six carbon atoms. These shortened dicarboxylic acids can undergo two processes: be moved to mitochondrion and be completely β-oxidized producing succinate or be excreted as a urine ingredient [13].

Dicarboxylic acids as ω-oxidation products, positively influence oxidative phosphorylation and thus supply necessary energy for cell repair and proliferation. Different derivatives of dicarboxylic acids play an important role in cell signaling both inside of the cell and between the cells. They are essential for: ionic channel activity and transporters, lipogenesis, mitogenesis, activation of tyrosine kinases and phosphatidylinositol 3-kinase pathways. Ω-hydroxylated fatty acids can be metabolized and may serve as a source of energy for lipogenesis, structural lipids biosynthesis and for the biosynthesis of fatty acids, which act as a regulators of hormonal receptors in the cell nucleus [14].

Traumatic acid and traumatin are plant wound-healing hormones causing cicatrization. Available data shows that TA stimulates cell proliferation and biochemical processes connected with cell division, like: protein, nucleic acid and photosynthetic dye biosynthesis and photosynthesis intensity. However, TA activity in human and animals cells has not been studied. The stimulatory effect of TA at the molecular level in plant and animal cells is probably similar to the activity of non-esterified fatty acids. It can be assumed that TA, which is an oxygen derivative of unsaturated fatty acids, can act in human cells, e.g., fibroblasts in the same way it acts at the molecular level in unsaturated fatty acids, non-esterified (free) fatty acids and protein-bound fatty acids.

## Materials and Methods

### Chemicals and Reagents

All the reagents: traumatic acid, phosphate-buffer saline (PBS), DMEM, FBS, 5,5′-dithiobis (2-nitrobenzoic acid; Ellman’s reagent), hydrogen peroxide, NADPH, meta-phosphoric acid (MPA), SDS (Sodium Dodecyl Sulphate), TCA (Trichloroacetic acid), TBA (Thiobarbituric acid) and the GPX Celluar Assay Kit were purchased from Sigma (St. Louis, MO, USA). The Glutathione Assay Kit was obtained from Merck. All other reagents and solvents were of analytical grade. Tissue culture dishes and flasks were purchased from Sarstedt.

### Cell Culture

TA's influence on tested parameters was examined in normal human skin fibroblasts in physiological conditions, without any stress factors. The fibroblast cell line was maintained in DMEM supplemented with 10 % FBS at 37 °C in a humified atmosphere of 5 % CO_2_ in air. Fibroblasts at a density of 1 × 10^5^ cells/ml were incubated with or without the test compound in tissue culture in six-well plates in 2 ml of culture medium. The cell number was calculated at TA concentrations of 10^−4^, 10^−5^, 10^−6^ and 10^−7^ M. Collagen content in cells and medium, total protein content, SDS-PAGE and basic oxidative stress parameters such as: antioxidant enzyme activity, reduced glutathione and SH-group content and lipid peroxidation were studied at two TA concentrations: 10^−5^ and 10^−6^ M.

### Chemical Treatment of Cells

Traumatic acid was stored in a refrigerator (below −4 °C), protected from light and was added to the cultured cells for a final concentration in the range of 10^−5^ to 10^−6^ M. The control cells were incubated without the test compound.

### Estimation of the Cell Number

The number of fibroblasts was estimated by direct counts of cells in the growth medium using a Bürker chamber.

In all performed assays, data were normalized to each other by measuring the total cell number to exclude the risk that variable cell numbers were lysed.

### Total Protein Content in Cells

After the homogenization of fibroblast cells and extraction in 0.1-M NaOH at 4 °C, the total protein content was calculated. The concentration of proteins was determined spectrophotometrically as per [15]. Folin phenol reagent with a protein kit calibrated with bovine serum albumin as the standard was used in the experiment. The absorbance of the extracts was measured spectrophotometrically at 750 nm [15].

### Electrophoresis SDS-PAGE

For SDS-PAGE, cells were collected by scraping in cold PBS, centrifuged and resuspended in 2 ml of PBS and stored at −70 °C. Cells were lysed by freezing and thawing to room temperature twice. To the aliquots of the cell lysates buffer was added (SDS, glycerol, mercapthanol, 0.5-M tris–HCl and bromothymol blue, pH 6.8). 25-μl samples of SDS:protein at a ratio 4:1 (v/v) were loaded onto a 10 % polyacrylamide gel containing 0.1 % SDS and the buffer system as per [16]. Gels were run at 20 °C at a constant current of 15 mA for approximately 4 h and then stained with Coomassie brilliant blue [16].

### Collagen Assay

This method is based on the spectrophotometrical observation that Sirius red in saturated picric acid selectively binds to fibrillar collagens (types I to V), specially to the Gly-X–Y fragment in the helical structure [17]. The assay measures soluble collagen (i.e., culture medium) and insoluble collagen. A working solution of collagen (250 μg/ml) was prepared by diluting stock with 0.5-M acetic acid. After preparing collagen standards, the samples were prepared. If assaying culture medium, the serum concentration must be no more than 5 %. This can be achieved either by switching the cells to 5 % serum prior o collecting the medium or by diluting the medium with water to 5 %. For assaying collagen in culture medium, 50–100-μl aliquots of standards, blank (medium) and samples were put into 1.5-ml-Eppendorf tubes. Subsequently, 1 ml of the dye solution was added and mixed gently at room temperature for 30 min. The samples were centrifuged at 10.000*g* for 5 min to pellet the collagen. The supernatant was carefully removed without disturbing the pellet. 1000 μl of 0.1-M HCl was added to each tube to remove unbound dye. Afterwards samples were centrifuged at 10.000*g* for 5 min to pellet the collagen, and 1000 μl 0.5-M NaOH was added to each tube; tubes were then vortexed vigorously to release the bound dye. The solutions were transferred to cuvettes and read at 540 nm. In assaying collagen in the cell pellet, the cell pellet was first extracted with 50 μl of 0.5-M acetic acid at 4 °C for several hours to overnight. After all mentioned above procedures, the solution was centrifuged at 2500*g* for 5 min to re-pellet any cell debris, and was then read at 540 nm.

### Enzyme Assays

For enzyme analysis, cells were rinsed with PBS at 4 °C and collected by scraping in cold PBS, centrifuged and resuspended in 1 ml of PBS and stored at −80 °C. Cells were lysed by freezing and thawing to room temperature twice. Aliquots of the cell lysates were collected for enzyme assays. Glutathione peroxidase (GPX, EC 1.11.1.9) activity was measured according to the method of Paglia and Valentine, using the GPX Cellular Activity Assay Kit (Sigma-Aldrich). An indirect determination method is based on the oxidation of glutathione (GSH) to oxidized glutathione (GSSG) catalyzed by GPX, which is then coupled to the recycling of GSSG back to GSH utilizing glutathione reductase (GR) and NADPH [18]. The decrease in NADPH absorbance measured at 340 nm during the oxidation of NADPH to NADP^+^ was indicative of GPX activity, since GPX is the rate-limiting factor of the coupled reactions. Catalase (CAT, EC 1.11.1.6) activity was measured spectrophotometrically at 240 nm by monitoring the decline in H_2_O_2_ in the presence of cellular lysates [19]. Activity was calculated using the rate of change per minute and the molar extinction coefficient (*λ*_240_ = 47) of H_2_O_2_. Glutathione reductase (GR, EC 1.8.1.7) activity was determined by the method reported by Mize and Langdon [20]. The reaction mixture consists of KCl in phosphate buffer (pH 7.0), GSSG, aliquots of the cell lysates and NADPH. The consumption of NADPH was monitored spectrophotometrically at 340 nm. Enzyme activity was calculated using the molar extinction coefficient of 6.22 mM/cm.

### Glutathione Assay

Cells collected for glutathione determination were rinsed with PBS at 4 °C and scraped from Petri dishes (between 1 and 5 × 10^6^/plate). The cells were then resuspended in 1 ml of cold PBS and lysed by freezing and thawing to room temperature twice. Subsequently, 1 ml of MPA (meta-phosphoric acid) working solution was added. Cells were incubated at 4 °C for 1 h and centrifuged at 13,500*g* for 10 min. The upper clear aqueous layer was used for the assay. Reduced glutathione (GSH) was determined by using the Glutathione Assay Kit (Merck). In this assay, chromophoric thione was obtained with a maximal absorbance at 400 nm.

### Determination of SH Groups

For the determination of total content of SH groups in fibroblasts, cells were washed twice with PBS (pH 7.4; 4 °C) and dispersed by scraping. The cells were counted, resuspended in 1 ml of PBS and collected by centrifugation at 5000*g* for 10 min. The pellet was resuspended in 1 ml of 0.5-M phosphate buffer (pH 7.8), containing 0.1 % SDS. Then, 25 μl Ellman’s reagent (5 mM) was added and the thiol groups were measured spectrophotometrically at 412 nm using the molar extinction coefficient of 13.6 mM^−1^ cm^−1^.

### Determination of TBARS

The level of TBA-reactive species (TBARS) as membrane lipid peroxidation markers was measured using the method of Rice-Evans *et al*. [21]. The cells were washed with PBS (pH 7.4), scraped from Petri dishes and resuspended in 1 ml of PBS (between 1 and 5 × 10^6^/plate). TCA (15 %, 1 ml) and TBA (0.37 %, 1 ml) were added to 1 ml of the cell suspension and mixed. This mixture was submerged in a boiling water bath for 10 min and the concentration of TBARS was assessed spectrophotometrically at 532 nm using the extinction coefficient of 156 mM/cm.

### Statistical Analysis

For parametric data, one-way analysis of variance (ANOVA) followed by Tukey's test was applied. Results from five independent experiments were expressed as mean ± standard deviation (SD) of the mean for parametric data. Statistical significance was considered when *p* ≤ 0.05. Statistica 12.5 was used.

## Results

### Cell Number

**Fig. 3:**
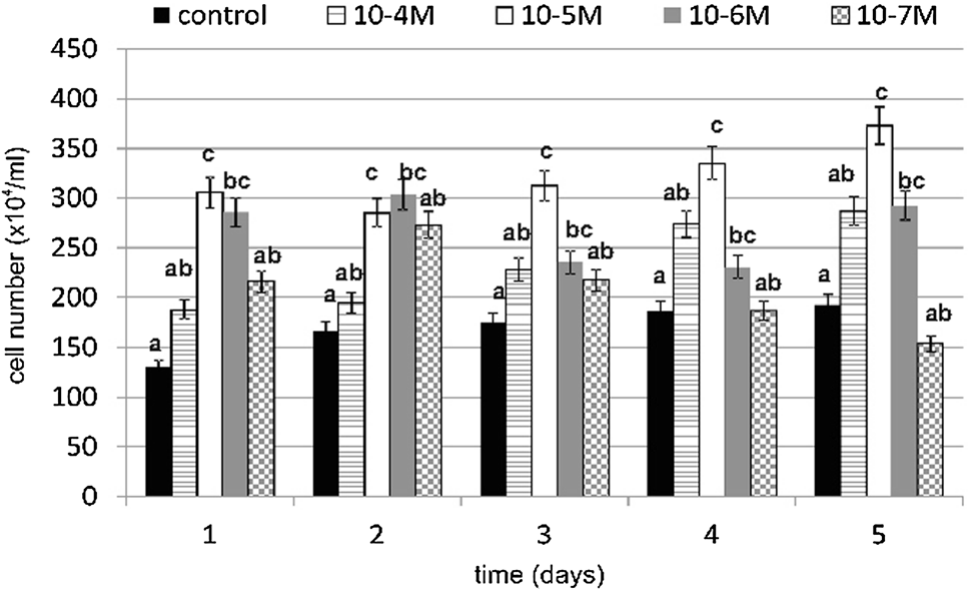
The effect of various concentrations of TA on fibroblast cell number during 5-day incubation (*n* = 5). Data are presented as the mean ± SD. *Different letters* (*a*, *b*, *c*) indicate statistical differences (≤0.05) estimated by Tukey's test

TA caused a significant increase in cell number, especially on day 1 at a concentration of 10^−5^ M (Fig. 3). Our results showed an increase of 133 % compared to the untreated control cells. However, treatment at a TA concentration of 10^−6^ M resulted in an increase of 118 % compared to the control cells. Statistically significant increases in cell number in concentrations of 10^−5^ and 10^−6^ M TA were observed.

### Total Protein Content in Cells

**Fig. 4:**
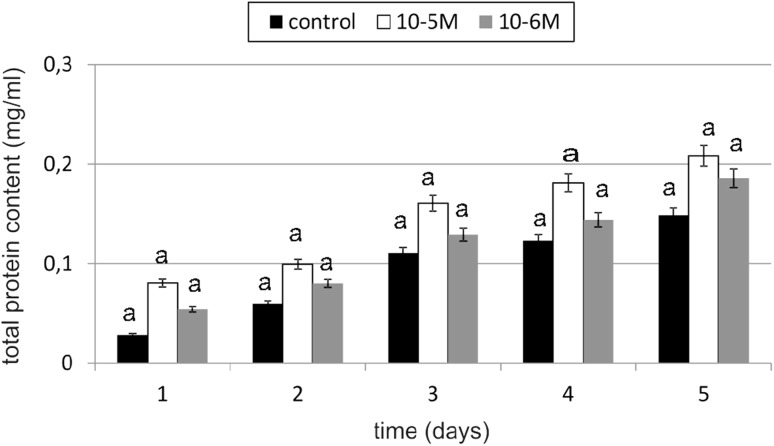
The effect of selected concentrations of TA on the total protein content of fibroblast cells during a 5-day incubation (*n* = 5). Data are presented as the mean ± SD. The *same letter* (*a*) indicates no statistical difference (≤0.05) estimated by Tukey's test

An increase in total protein content of 183 % compared to the control was observed in TA-treated cells at concentration of 10^−5^ M on day 1 (Fig. 4). TA at a concentration of 10^−6^ M also increased total protein content by 90 % compared to the untreated control cells. Any decline below the level of control was not observed. Observed changes were statistically insignificant.

### Electrophoresis SDS-PAGE

**Fig. 5:**
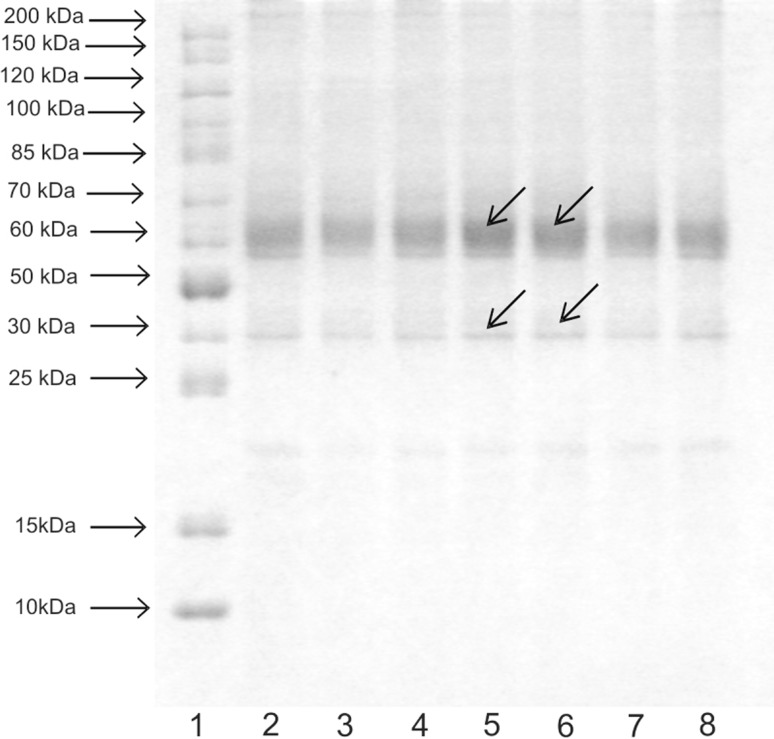
SDS-PAGE of the total proteins isolated from fibroblasts cultivated in the presence of 10^−5^-M and 10^−6^-M TA compared to the control culture (*1* marker, *2* control, *3* 10^−5^-M TA-treated cells day 3, *4* 10^−6^-M TA-treated cells day 3, *5* 10^−5^-M TA-treated cells day 4, *6* 10^−6^-M TA-treated cells day 4, *7* 10^−5^-M TA-treated cells day 5, *8* 10^−6^-M TA-treated cells day 5)

After comparing the picture for the control with the picture for TA-treated cells, there is a visible difference especially concerning proteins with molecular weights of 30 kDa and approximately 64 kDa (Fig. 5). In cells treated with TA, on day 4, these proteins demonstrated noticeably darker bands. Other proteins are on the same level as in the control.

### Collagen Content in Cells and Medium

**Fig. 6:**
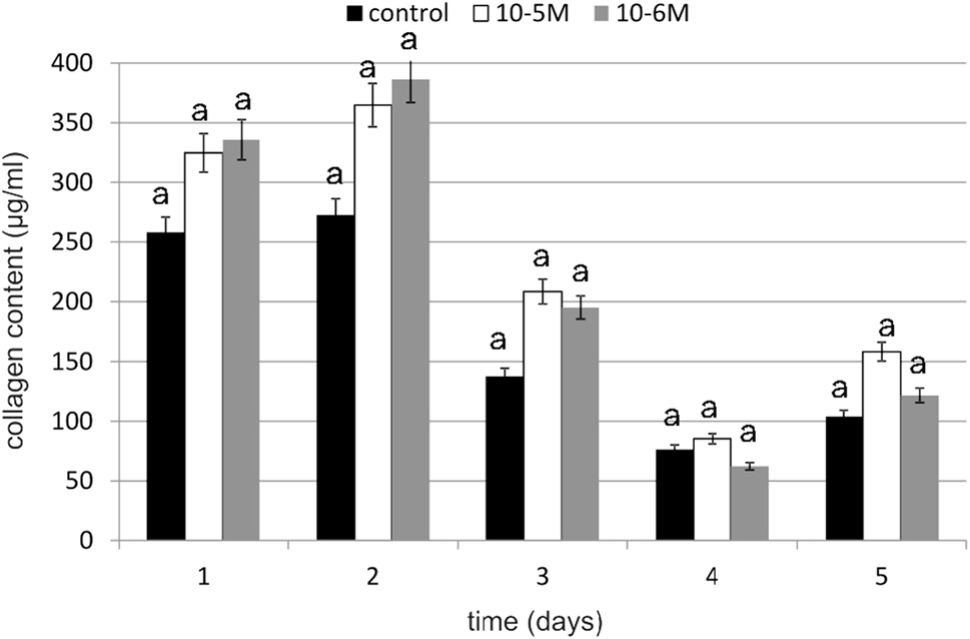
The effect of selected concentrations of TA on collagen content in cells during a 5-day incubation (*n* = 5). Data are presented as the mean ± SD. The *same letter* (*a*) indicates no statistical difference (≤0.05) estimated by Tukey's test

**Fig. 7:**
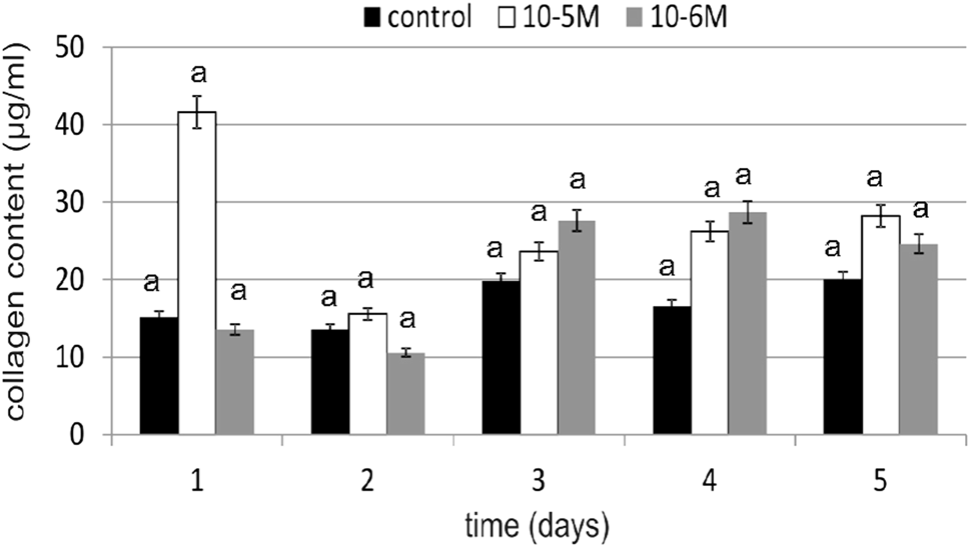
The effect of selected concentrations of TA on collagen content in medium during a 5-day incubation (*n* = 5). Data are presented as the mean ± SD. The *same letter* (*a*) indicates no statistical difference (≤0.05) estimated by Tukey's test

Collagen is the main structural component of connective tissue, that maintains the stability of organs and supports their structural integrity. It is synthesized mainly by fibroblasts. Because the intensity of this biosynthesis decreases with age, it is important to find an effective and safe substance that will stimulate it. Under the influence of TA, the amount produced and secreted to medium collagen was higher (Figs. 6, 7). On day 1, an increase in collagen content compared to the control was observed (at 10^−5^ M). At 10^−6^ M on day 4, TA caused an increase in collagen content of 72 % compared to the control. Stimulation of collagen biosynthesis in TA-treated fibroblasts was observed on day 3. On day 1, at 10^−5^ M, TA caused an increase of 51 % in collagen content in cells compared to the control, while at 10^−6^ M, it was a little less effective, resulting in an increase of 41 %. Obtained results of the TA concentration influence on collagen biosynthesis were statistically insignificant.

### Antioxidative Enzyme Activity

**Fig. 8:**
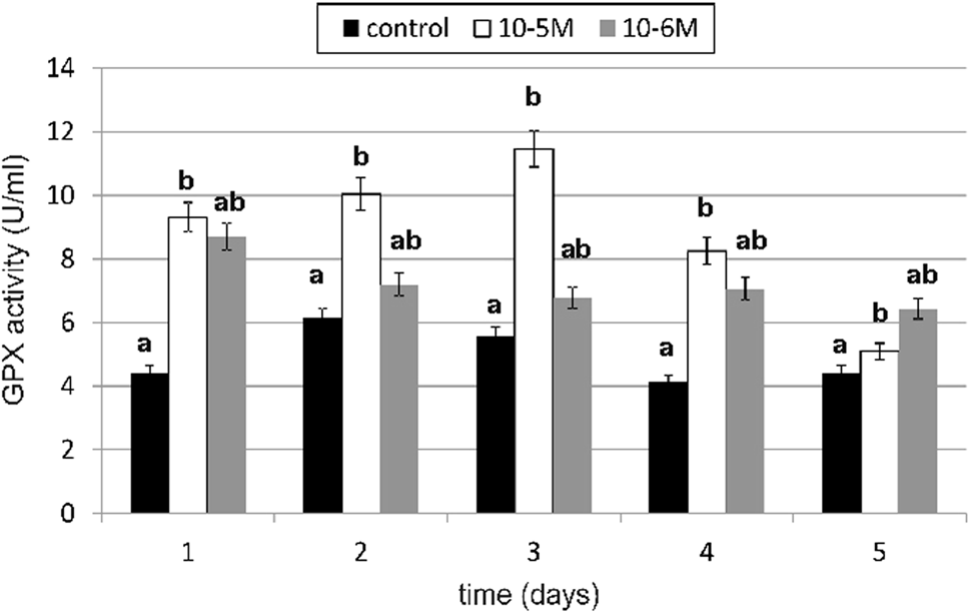
The effect of selected concentrations of TA on GPX activity during a 5-day incubation (*n* = 5). Data are presented as the mean ± SD. *Different letters* (*a*, *b*) indicate statistical difference (≤0.05) estimated by Tukey's test

**Fig. 9:**
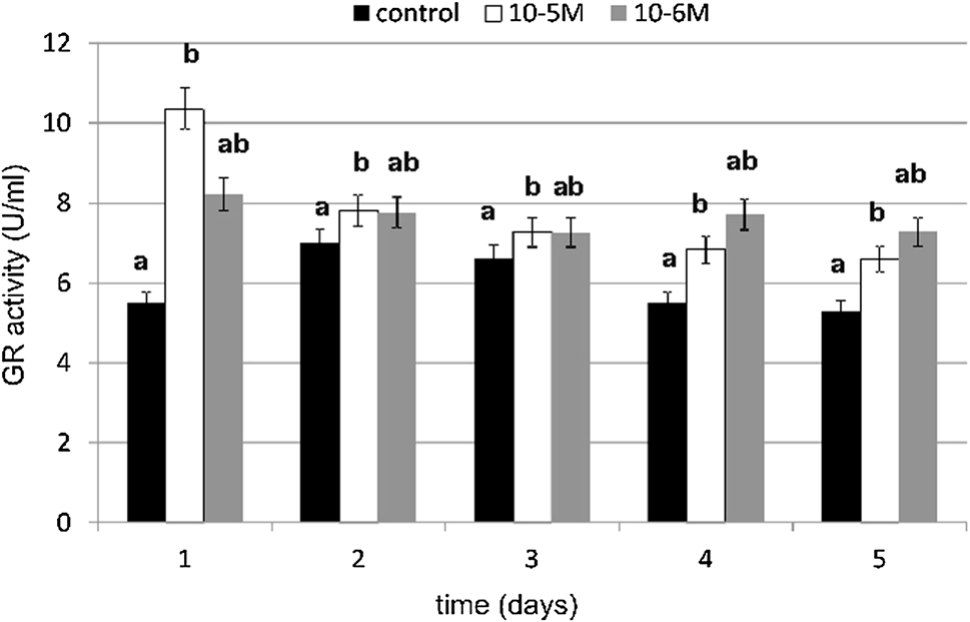
The effect of selected concentrations of TA on GR activity during a 5-day incubation (*n* = 5). Data are presented as the mean ± SD. *Different letters* (*a*, *b*) indicate statistical difference (≤0.05) estimated by Tukey's test

**Fig. 10:**
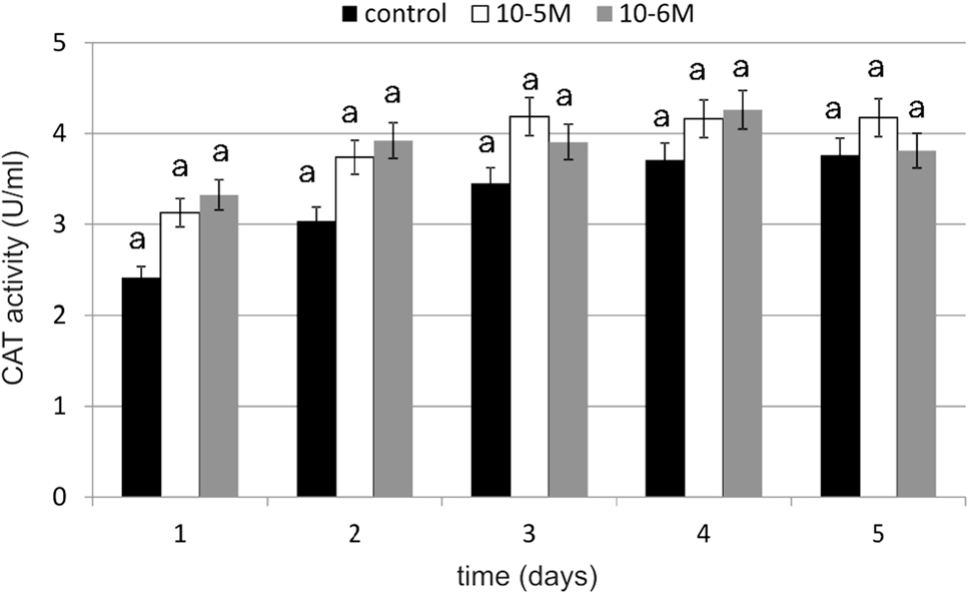
The effect of selected concentrations of TA on CAT activity during a 5-day incubation (*n* = 5). Data are presented as the mean ± SD. The *same letter* (*a*) indicates no statistical difference (≤0.05) estimated by Tukey's test

The primary enzymatic antioxidant defense system, including glutathione peroxidase (GPX), glutathione reductase (GR) and catalase (CAT), is the first-line defense to detoxify reactive oxygen species (ROS). A significant increase in GPX activity under the influence of TA was observed on days 1, 3 and 4 (Fig. 8). TA at a concentration of 10^−5^ M increased GPX activity by 111 % compared to the control, which was statistically relevant. At a concentration of 10^−6^ M, TA was slightly weaker in enzyme activity stimulation. It increased GPX activity by 97 % compared to the control. Similarly to GPX, a significant increase in GR activity was observed at both tested concentrations (Fig. 9). The GR activity was the highest at a 10^−5^-M concentration (by 88 % compared to the control) on day 1. A slight decrease in tested enzyme activity was observed on day 2. In next days we observed increase in enzyme activity again. The influence of TA at a 10^−5^-M concentration on GR activity was statistically significant. The increase in GR activity at a 10^−6^-M concentration wasn’t as high as it was at a 10^−5^-M concentration, but the activity was maintained on a higher level on day 4 and 5 (respectively, by 40 and by 37 %). Our results shows that TA increased CAT activity especially on day 1 (Fig. 10). At a 10^−5^-M concentration of TA, we observed an increase of 29 % compared to the untreated cells, and at a 10^−6^-M concentration of TA, we observed an increase of 37 % compared to the control. No decreases were observed in CAT activity in both tested concentrations compared to the control. The obtained results regarding TA influence on CAT activity were statistically insignificant.

### Reduced Glutathione Content

**Fig. 11:**
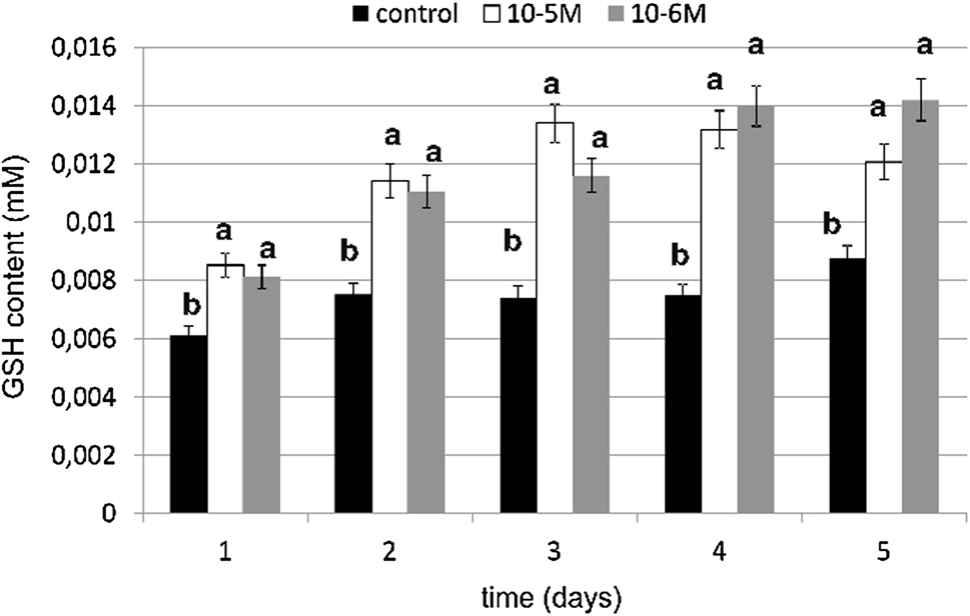
The effect of selected concentrations of TA on GSH content during a 5-day incubation (*n* = 5). Data are presented as the mean ± SD. *Different letters* (*a*, *b*) indicate statistical difference (≤0.05) estimated by Tukey's test

Reduced glutathione (GSH) is a fundamental low-molecular mass antioxidant that plays an important role in many biological processes and its determination is a very useful tool in studying oxidative stress. An especially high content of reduced glutathione (GSH) was observed at a 10^−6^-M concentration and it was an increase of 86 % as compared to the control (Fig. 11). However, at a 10^−5^-M concentration of TA we observed an increase of 80 % compared to the untreated cells. No decreases below the control level were observed. TA in both tested concentrations caused an increase of 80 % compared to the control. Reduced glutathione level was relatively high under TA influence in both tested concentrations. The obtained results regarding the influence of TA on GSH content were statistically significant for both tested concentrations. These data shows stimulatory effect of TA on GSH biosynthesis in fibroblasts.

### SH Groups Content

**Fig. 12:**
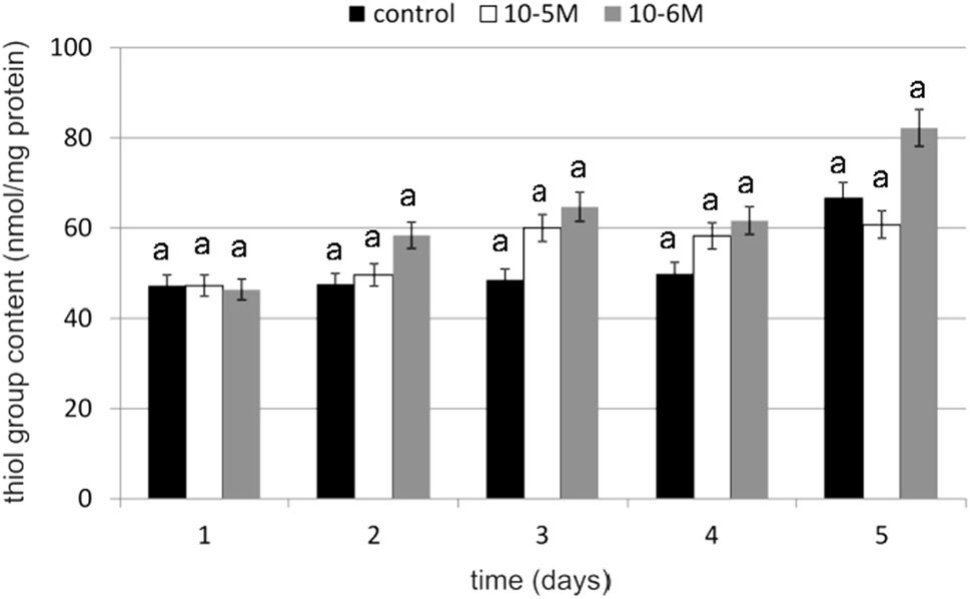
The effect of selected concentrations of TA on SH group content during a 5-day incubation (*n* = 5). Data are presented as the mean ± SD. The *same letter* (*a*) indicates no statistical difference (≤0.05) estimated by Tukey's test

To determine the oxidation of the SH group's spectrophotometric assay with Ellman’s reagent was used. Thiol group content was evaluated as a marker of protein oxidation. An increase in thiol group content of 33 % compared to untreated control cells was observed on day 3 at a TA concentration of 10^−6^ M (Fig. 12). Exposure to TA resulted in an approximately 23 % increase in the total cellular content of the thiol groups on day 3. No decreases in SH group content below the control level were observed. Observed changes were statistically insignificant. These data indicate that the tested cytokinin does not cause oxidative damage in proteins.

### Lipid Peroxidation

**Fig. 13:**
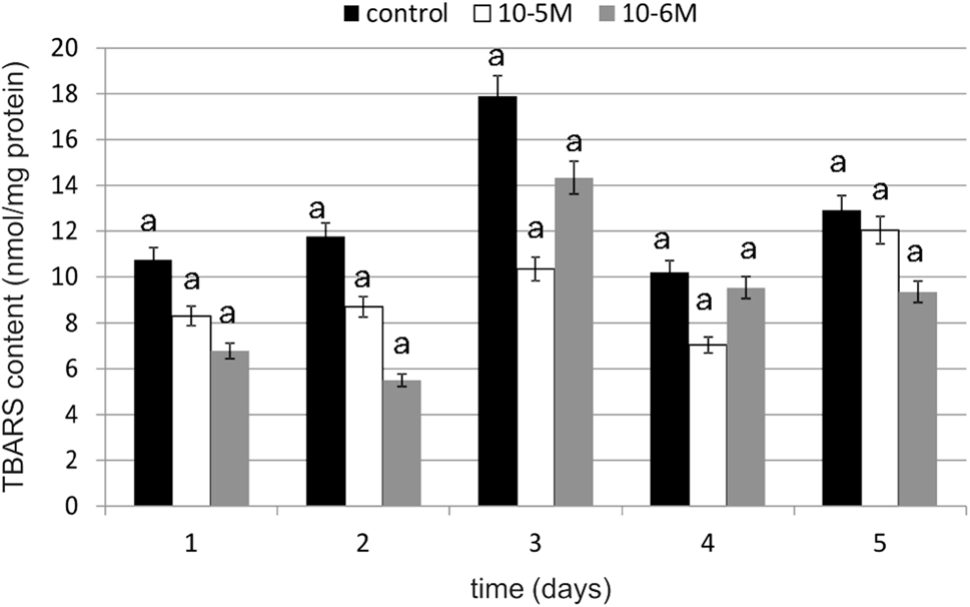
The effect of selected concentrations of TA on TBARS content during a 5-day incubation (*n* = 5). Data are presented as the mean ± SD. The *same letter* (*a*) indicates no statistical difference (≤0.05) estimated by Tukey's test

Lipid peroxidation is a deleterious process that leads to structural modifications of complex lipid protein assemblies, such as biomembranes and lipoproteins and is usually associated with cellular malfunction. TBARS content was measured as an index of lipid peroxidation. The results showed a difference between TBARS levels in the control and TA-treated cells; however, these changes were statistically insignificant (Fig. 13). The addition of this cytokinin to the cells induced reduction in TBARS content compared to the control. At a TA concentration of 10^−5^ M, a decrease in TBARS content of 42 % compared to the control was observed on day 3. However, TA was most effective at a concentration of 10^−6^ M, resulting in a decrease of 53 % compared to the control on day 2. The obtained results suggest that the cytokinin demonstrates protective properties against TBARS production and, as a consequence, decreases membrane phospholipid peroxidation.

## Discussion

Cytokinins are one of the major groups of plant hormones. Their biochemical transformations and molecular mechanism of their action in plant cells are quite well-known, but there is significant lack of information concerning cytokinins' influence on animal and human cells growth and metabolism. Human skin fibroblast *in vitro* culture is an appropriate research model, which allows examination of biologically active compounds' influences on basic biochemical parameters and morphological changes in dermis. Cytokinins, particularly TA, have not been researched as potential therapeutical substances, and, therefore, in this report we’re trying, for the first time, to present TA influence on cell number, total protein content, collagen content, and basic oxidative stress parameters, such as antioxidative enzymes activity, reduced glutathione content, thiol groups content and lipid peroxidation.

The effect of TA on human and animal cells has not previously been investigated and described, and, therefore, the obtained results can be referred only to a few literature data concerning the action of TA in plants. TA is a wound-healing hormone, which stimulates cell division in wounded plant tissues; thus, it appears that it will act similarly in human cells. Our results revealed that it significantly stimulates fibroblasts proliferation, causing a considerable rise in cell number, especially at concentrations of 10^−5^ and 10^−6^ M. In a like manner, TA acts on cell proliferation in plants, in particular, in unicellular algae *Chlorella vulgaris* [22]. The molecular mechanism of TA action and its impact on the basic metabolic and physiological processes, even in plants, is not yet completely understood. It was found that the tested compound increases or decreases the rate of protein phosphorylation depending on their molecular weight, which results from the phytohormone influence on the activity of particular protein kinases and phosphatases. If TA binds to a specific receptor in the cell membrane, it may thus activate a cascade of signal transduction and stimulate mitotic division of cells. This compound can also act like other compounds analogous in structural terms, e.g., dicarboxylic acids (DCA). DCA are produced as a result of ω-oxidation and they may cause, similar to plant cells, an increase in oxidative phosphorylation and their role could rely on an energy supply for cell proliferation and regeneration [14]. It can be concluded that the TA stimulation of cells proliferation results from the kinases cascade activation or from an appropriate energy level in cells, as with the other DCA. DCA-analogous compounds and DCA derivatives play an important role in cell signaling. They are essential for the proper functioning and activity of ionic channels and lipogenesis. They activate tyrosine kinases and phosphatidylinositol 3-kinase, which phosphorylate phosphatidylinositol in the cell membrane. Then, the anchorage for intracellular, signaling proteins are created, e.g., for protein kinase B, which phosphorylates serine and threonine in proteins and, therefore, is crucial for cell life and proliferation. Inadequate protein phosphorylation by the protein kinase B inhibits its pro-apoptotic properties, and prevents the cell entering the path of apoptosis [14]. TA as a dicarboxylic acid can also act by the molecular mechanisms described above.

In our study, TA affected the morphological organization of fibroblasts, maintaining their fusiform, elongated shape and their migration. In the Wound Assay test, it intensively accelerated the cicatrisation of artificially created “wounds” (unpublished data). The cells under the influence of TA at a concentration of 10^−6^ M migrated very quickly and inhabited an area of culture dish; this may be related to the supply of energy required for cell migration. According to the literature, omega-hydroxy fatty acids can be metabolized and used for energy production [14]. Their effect on collagen is related with cell movement, because fibroblasts play an important role in the formation of collagen, by participating in the transportation of collagen in membranous structures or in the formation of fiber networks. We observed TA's effect on the biosynthesis of collagen and cellular proteins. It stimulated total protein biosynthesis and total protein content in cells, although the effect was statistically insignificant. TA had a stimulating effect on protein biosynthesis in the first 2 days of the experiment at concentrations of 10^−5^ and 10^−6^ M. The increase in total protein content after TA stimulation was likewise observed by other authors [8] in *Chlorella vulgaris* algae cultures. DCA and most likely TA affect the activity of fatty acid protein transporters. Until recently it was thought that the fatty acids enter into cells via simple diffusion, in accordance with the concentration gradient. It has been demonstrated, however, the presence of various fatty acid transporter protein in various cells and tissues. Results of the electrophoresis revealed the presence of two intensively stained bands corresponding to the MWs of 63 kDa. The bands probably originated from one of the fatty acid transport proteins (FATPs). FATPs exist in six isoforms, but in fibroblasts, only one of them is detected (FATP1). It consists of 646 amino acids and has a molecular weight 63 kDa [23]. It was also shown that FATP1 has a long-chain fatty acid synthetase internal activity and it plays an important role in fatty acid transformation into acylo-CoA which occurs in cytoplasm [23–25].

TA influence on the collagen content in fibroblasts is quite unclear. We observed both increases and decreases in tested protein content during experiment. TA was especially active at a concentration of 10^−5^ M at which it caused increase in examined protein content of 173 % compared to the control on day 1. The next day, a slight decline was observed (of 14 % compared to the control), and, at the same time, we noticed an increase in collagen content in cells. On day 3, a decrease in medium and, at the same time, an increase in cells was observed. However, on day 4, a decrease in collagen content in fibroblasts was observed and, simultaneously, an increase in medium was noticed. It may indicate that collagen is transported from cells to medium. Because the research on the impact of TA on the human skin fibroblasts were not yet conducted, it is not known which of the molecular mechanisms causing the observed changes in the collagen content in the cells is of the main importance. There is a possibility of TA binding to nuclear receptors and, thus, enhancing the expression of collagen encoding genes. TA belongs to the fatty acids and may, like other NEFA (non-esterified fatty acids), be involved in at least two signal transduction pathways from the hydrophilic membrane surface to the hydrophobic interior of the nucleus. NEFA are present inside of the cell for a very short period of time and in specific locations, therefore proving their presence in the cell or outside the cell in non-estrified and protein unbound forms is almost impossible. The NEFA pool, especially the essential fatty acid pool, is affected by a variety of exogenous or endogenous factors. One of the exogenous factor is, e.g., nutrition. Several studies have shown that the fatty acid profile in a cell depends on their participation in the diet [26]. The endogenous factor that affects NEFA pool is the presence of lipases, acyltransferases and transacylases. These enzymes are activated by a variety of signaling compounds (streroids, bacteria toxins, thrombin, neurotransmitters). They are active against a large range of substrates, releasing or incorporating into their structure the saturated and unsaturated fatty acids. Both fatty acids and, therefore, probably TA may perform various biological functions at the cellular level associated with the proliferation and differentiation of cells. The different effects on the cells and functioning of the entire organism is associated with their heterogeneous structure, the concentration and an association with other chemical agents. The amount of fatty acids in the cells also depends on their metabolic transformations as a result of which other signaling molecules, e.g., prostaglandins and leukotrienes, are synthesized. As previously mentioned, they are also bound by specific proteins (albumin, alphafoetoprotein, FABP [fatty acid binding proteins] and LDL [low density] proteins). Available data show that NEFA, which act as second messengers or modulators in the cell network, are under the control of the cell or plasma FABP [26].

NEFA can act as second messengers or modulators of signal transmissions from the extracellular environment through the various, associated with each other signal transduction pathways, while not subject to further catabolic transformations to the oxidized metabolites. Unsaturated fatty acids, as messenger molecules, stimulate the activity of protein kinase C by the diacylglycerol analogous action or by the release of calcium ions from the endoplasmic reticulum via a mechanism which is, in this case, independent of the rotation of phosphoinositol [27, 28]. The phosphorylation process, which was already mentioned, plays here an important role. The activation of the phosphorylation by the fatty acids and probably also by TA may directly affect the gene transcription via phosphorylation of an essential transcription factor or indirectly by the phosphorylation of an additional factors that are involved in regulation of gene transcription. The above-mentioned protein kinase C (PKC) activation is an example. PKC, like other protein kinases, phosphorylates transcription-activating factors, which in turn interact with the cis-active transcription control elements to regulate the expression of associated genes [29]. It is worth mentioning that the unique characteristic feature of FABP is their phosphorylation catalyzed by the tyrosine kinases which occurs at the N-terminal end [30].

NEFA act as modulatory molecules when in a reversible manner; in a specific place and for a very short period of time, they act as second messengers of the cellular signals. Furthermore, they can strengthen, weaken or completely change the signal caused by second messengers. They are positive or negative modulators of the activity of several members of the v-erbA super family of receptors (estrogen, glucocorticoid, progestin, androgens, retinoids and peroxisome proliferator-activated receptors), which are transcription factors. Membrane and nuclear signal transduction pathways are converged and NEFA are associated with it. They can act through two molecular mechanisms [31].

The first one is the transconformation of protein transcription factors via their phosphorylation and NEFA binding. These transcription factors can activate the nuclear v-erbA superfamily receptors or membrane signal transduction associated with oncogenic agents. Transduction via membrane peptides induces transcription complex AP-1 which consists of c-jun and c-fos proteins. This complex is activated by various extracellular signal peptides (cytokines, growth factors and tumor promoters) and it plays an important and major role in cell proliferation. The phosphorylation of these factors is under the influence of fatty acids that activate protein kinases or inhibit certain dephosphorylases. Phosphorylation and dephosphorylation of the transcription factors and the NEFA bonding to these factors (both reversible and irreversible) may affect protein activity by inducing conformational changes in them, and causing the change of their electrostatic properties that modify the action of transcription factors. The activity of transcription factors may be regulated positively or negatively by phosphorylation and binding of fatty acids. The effect may be exerted on both the ability to DNA binding and transactivation functions. However, the effect of fatty acids on transcriptional regulation by phosphorylation and dephosphorylation still requires many researches supporting and determining the manner in which the fatty acids affect cellular proliferation and differentiation [32–34].

The second mechanism is the interference of nuclear factors with the membrane, oncogenic factors associated with transmembrane signal transduction. At the gene level there is a wide variety of different types of connections between the transcription factors and other supporting proteins. The final conformation of the dimmers and associated molecules will condition the specificity of the DNA-binding elements and by a consequence the gene expression can be positively or negatively influenced. Fatty acids and TA can, therefore, directly, by attaching or acetylation, or indirectly, by phosphorylation or FABP binding, impart effects on the expression of specific genes [27].

The aging of an organism, including the skin, can be either the result as well as a cause of increased production of ROS, which are products of the reduction or stirring of the molecular oxygen, causing oxidative stress. The free radical theory of aging claims that there is a correlation between aging, oxygen metabolism and free radicals biosynthesis. Oxidative stress is mostly connected with human skin, which possesses the largest surface area in the human body and serves as the protective layer for internal organs. The skin structure, rich in lipids, proteins and DNA, is designed to give biochemical and physical protection and it is equipped with a large number of defense mechanisms. Because of the functions and localization in human body, skin runs the risk of oxidative stress the most of all tissues. Due to the high occurrence of potential biological targets for free radical reactions, skin is the most susceptible to oxidative damage. It is also exposed to a variety of damaging species, which come from outer environment, from endogenous sources or from the skin itself [35]. The exogenous sources are: air pollutants, natural deleterious gases (e.g., ozone, hyperbaric oxygene), ionizing and non-ionizing irradiation, pathogenic viruses, bacteria, chemicals and toxins. There is also another important factor: physical damage to the skin, e.g., burns and wounds [35, 36]. The endogenous sources are mostly enzymes which may produce ROS, e.g., xanthine oxidase and nitric oxide synthase or lipooxygenases and cyclooxygenases, which play important roles during eicosanoid metabolism. An example of an enzyme which can produce free radicals directly in the skin is nitric oxide synthase. An ROS source may also be activated white cells such as neutrophils, which undergo respiratory burst resulting in the release of an efflux of ROS and other compounds which may cause oxidative damage in the skin tissue (inflammatory process). The other source of free radicals are ischemic and post-ischemic processes that usually cause an overproduction of ROS [35, 37].

The components of a skin antioxidative system can be divided into two parts: enzymatic and non-enzymatic. The group of antioxidant enzymes includes the enzymes, e.g., superoxide dismutase, catalase, glutathione peroxidase and some supporting enzymes, like glutathione reductase. According to the literature data, catalase and glutathione peroxidase are much more effective in fibroblast protection against oxidative stress than superoxide dismutase. Moreover, superoxide dismutase overexpression causes a higher sensitivity to oxidative stress in cells and it doesn’t protect against that factor [38, 39]. Reduced glutathione (GSH), which is cosubstrate for glutathione peroxidase, plays an important role in the antioxidative defense system in cells. Glutathione disulfide (GSSG) which is produced in reaction catalyzed by GPX, is reduced by GR (with NADPH) to GSSG. The main function of GSH is maintaining protein SH groups in a reduced state. GSH has many more functions, e.g., removal of harmful products of lipid peroxidation, antioxidative enzymes cofactor, antioxidants recycling (e.g., ascorbic acid), protection against inflammatory response, influences on transcriptional factors (NF-κB and AP-1), regulation of the cell proliferation and protection against apoptosis. Probably during the aging processes, the activity of antioxidative enzymes, which reduce GSSG, decreases [40, 41].

Our results show that under the influence of TA, activity of antioxidative enzymes significantly increases right after 24 h of incubation with tested compound. Any decrease below enzyme activity was not observed compared to control cells. GPX activity was on the highest level on the first day of cultivation and on the following days, it was also high, especially on day 3 and 4. GR also had the highest activity on day 1. On the following days, we observed a slight decrease in the enzyme activity, but it was still higher activity as compared to the control. Obtained results were statistically significant. Similar results were obtained for the catalase, but, in this case, the activity increase wasn’t as high as compared to the other two enzymes and it was not statistically significant. Our results show that TA stimulates the activity of investigated enzymes. Thus far there is no literature data concerning TA's influence on antioxidative enzyme activity. The influence of fatty acids on oxidative stress and free radical generation also hasn’t been investigated till now. Fatty acids, which include TA, are highly energetic compounds which may influence ROS metabolism in many different ways. Fatty acid catabolism in mitochondrion (β-oxidation and acetyl CoA degradation through tricarboxylic acid cycle) delivers reducing agents to redox reactions. An increase in electron efflux through an electron transport chain is connected with increased H_2_O_2_ formation [42]. On the other hand, fatty acids may reduce H_2_O_2_ formation in mitochondrion through UCP proteins and adenine nucleotide translocase activation [43]. According to Duval *et al*. [44], fatty acids enhance antioxidant defenses against mitochondrial oxidative stress through epidermal growth factor receptor (EGFR)-dependent GPX activation. The activity of GPX is known to be regulated by various physiological or pathological factors in cells. For example, it can be down-regulated by oxidative stress, selenium deficiency and adduct formation through glycation or lipoxydation. On the contrary, GPX activity can be up-regulated by ischaemia, hypertension, traumatic injury, laminar shear stress, endotoxin, obesity and physical training. GPX gene expression is in part stimulated by ROS and mediated by nuclear factor-κB. But according to Duval *et al*. [44], fatty acids induce GPX activity not by gene induction, but rather by signal-dependent regulation of enzyme activity. It results from the fact that GPX activity was blocked by tyrosine kinase inhibitors. Fatty acids activate many various signaling pathways, such as protein kinase C, phosphoinositide 3-kinase, mitogen-activated protein kinase and EGFR. Therefore, we suspect that especially GPX, GR and also CAT activation under the influence of TA may act through the protein kinase activation pathway, which may initiate a signal cascade, the result of which is an observed increase in antioxidative enzyme activity. EGFR may be also a factor which is responsible for the observed increase in enzyme activity under the influence of TA, because EGFR regulates many important cells processes, including migration, proliferation and differentiation in cells. If TA, similar to other unsaturated fatty acids, activated EGFR, it would be possible that this is the signal transduction pathway which TA uses for activation of many metabolic functions in fibroblasts. EGFR is a transmembrane glycoprotein that belongs to the protein kinase superfamily. This protein is a receptor for the epidermal growth factor family members such as: EGF, heparin-binding EGF, TNF-α, amphiregulin, betacellulin and others. Its activity is influenced and regulated by a variety of unspecific stimuli, like: UV radiation, H_2_O_2_, oxidized lipoproteins, unsaturated fatty acids and their oxidation derivatives. The ligand binding process is followed by a series of metabolic and structural changes: the dimerization of EGFR, an increase in its intrinsic tyrosine kinase activity, and tyrosine residue undergoes autophosphorylation. In the structure of the C-terminal domain of EGFR, phosphotyrosines are localized. They serve as binding sites for SH2 domains of adaptors or enzymatic proteins including phospholipase Cγ1, GTPase-activating protein of p21ras (ras-GAP), SHP2, the p85 subunit of phosphatidylinositol 3-kinase SHC, Nck, c-*cbl* and GRB2-Sos. A consecutive step after GRB2-Sos complex activation is p21ras activation and it causes a kinase cascade which results in MAPK activation. The group of factors that may activate MAPK and ERK (extracellular-regulated kinase) include: receptors for growth factors, hormones, cytokines, G-protein-coupled receptors or stress factors (e.g., oxidative stress) [45]. According to Vacaresse *et al*. [45], unsaturated fatty acids may activate EGFR through induction of EGFR autophosphorylation and subsequent MAPK activation, and EGFR is considered as a primary target of unsaturated fatty acids. TA may, similar to oleic acid, bind to the EGFR domain (other than EFG binding domain) and activate the receptor. In terms of structure and properties, TA may interact with the hydrophobic domain, e.g., directly with the transmembrane domain or it may bind with EGFR after penetrating the lipid cell membrane.

The increase in investigated antioxidative enzyme activity and the significant increase in GSH content can be explained by the EGFR activation and kinases cascade. Unsaturated fatty acids activate protein kinase C and they activate a kinase cascade that may trigger appropriate genes [46]. Activated protein kinase C acts through a cell-signal cascade as a catalase activator [47]. TA may also activate protein kinase C and activate catalase in that way. Our results connected with GSH content in cells supports data concerning the influence of fatty acids on GSH content. In our investigations, GSH content in cell cultures stimulated with TA was significantly higher as compared to control cells. The increase was 86 % on day 4 at a TA concentration of 10^−6^ M. Obtained results revealed that both tested concentrations of TA have statistically significant effect on GSH content. A relatively high level of GSH under the influence of TA was correlated with high activity of GR. That also explains maintenance of a high level of SH groups in proteins. Our results are in agreement with literature data concerning fatty acids and the influence of their derivatives on reduced glutathione content in cells. According to Arab *et al*. [48], linoleic acid significantly induces GSH biosynthesis in fibroblasts without upsetting the redox balance in cells and without any induction of excess of lipid peroxidation. It stimulates enzymes connected with GSH metabolism, e.g., glutathione reductase, glutathione S-transferase and gammaglutamylcysteinyl ligase. Linoleic acid is a precursor in TA biosynthesis; therefore, we suppose that TA may act similarly and influence GSH content in an analogous manner. The observed significant increase in GSH content can be connected with the decrease in lipid peroxidation and membrane protein oxidation, because GSH plays an important role in maintaining protein thiol groups in a reduced form. We, likewise, observed a decrease in TBARS content, which is the most important lipid peroxidation marker. The increase in thiol groups under the influence of TA seems to confirm this thesis.

According to the literature, the is a strong connection between lipid peroxidation products and collagen biosynthesis in fibroblasts. The presence of the bonds between proteins and aldehydes (e.g., 4-HNE, TBARS, MDA) may increase the efficiency of the transcription of genes encoding collagen molecules. The above-mentioned aldehydes create bonds also with DNA and, therefore, they stimulate collagen gene expression. 4-Hydroxynonenal (4-HNE) has a major impact on cell growth and proliferation through the modulation of various signaling pathways. We did not analyze 4-HNE but we suppose that its content may change under the influence of TA similar to analyzed TBARS. Those changes may be connected with collagen biosynthesis and cell growth. 4-HNE growth-modifying activity was indicated in the HeLa cell line [49, 50]. In our experiment, we observed a slight relation between changes in cell proliferation and reactive aldehyde content. These chemical compounds act not only in abnormal conditions but, likewise, in physiological conditions, as in our study. This is in accordance with literature data which demonstrate that 4-HNE has a stimulating effect on cell growth not only in malignant cells but also in mesenchymal cells, such as bone [51]. It was also shown that 4-HNE is one of the very important products of n-6 polyunsaturated fatty acid peroxidation. The oxidation of multiple double bonds existing in polyunsaturated fatty acids by ROS causes the initiation of lipid peroxidation. As effects of this process, a diversity of reactive aldehydes arises and these aldehydes play an important role in human metabolism. 4-HNE, as one of the reactive aldehydes, influences cellular response to stress, causing an increase in antioxidant enzyme activity, such as catalase [52, 53]. Regarding the data obtained by Andrisic *et al*. in transgenic yeast, we assume that TA, as an unsaturated fatty acid (similar to polyunsaturated fatty acids), may cause metabolic changes leading to adaptation to oxidative stress, including an increase in catalase activity.

Traumatic acid can be a compound with a potential therapeutical application, because of its properties similar to unsaturated fatty acids. It can be a healthy substitute for fatty acids derived from animals tissues. Its beneficial antioxidative properties and its capacity to stimulate cell proliferation and protein kinase activity can be used for prevention and therapy of many blood circulation system diseases, including arterial hypertension and, likewise, for therapy of skin diseases connected with oxidative stress.

## Abbreviations

TA: Traumatic acid
GPX: Glutathione peroxidase
GR: Glutathione reductase
CAT: Catalase
GSH: Reduced glutathione
TBARS: TBA reactive species
LOX: Lipooxygenase
13-HPOT: 13-Hydroperoxylinolenic acid
13-HPOD: 13-Hydroperoxylinoleic acid
DCA: Dicarboxylic acid
HPL: Hydroperoxide lyase
LCFA: Long-chain fatty acids
MCFA: Medium-chain fatty acids
